# RAG-seq: NSR-primed and Transposase Tagmentation-mediated Strand-specific Total RNA Sequencing in Single Cells

**DOI:** 10.1093/gpbjnl/qzae072

**Published:** 2024-10-10

**Authors:** Ping Xu, Zhiheng Yuan, Xiaohua Lu, Peng Zhou, Ding Qiu, Zhenghao Qiao, Zhongcheng Zhou, Li Guan, Yongkang Jia, Xuan He, Ling Sun, Youzhong Wan, Ming Wang, Yang Yu

**Affiliations:** China-Japan Union Hospital of Jilin University, Jilin University, Changchun 130033, China; School of Life Sciences, Jilin University, Changchun 130012, China; Guangzhou Women and Children’s Medical Center, Guangzhou Medical University, Guangzhou 510623, China; Guangzhou Women and Children’s Medical Center, Guangzhou Medical University, Guangzhou 510623, China; Institute of Biophysics, Chinese Academy of Sciences, Beijing 100101, China; Guangzhou Women and Children’s Medical Center, Guangzhou Medical University, Guangzhou 510623, China; Guangzhou Women and Children’s Medical Center, Guangzhou Medical University, Guangzhou 510623, China; Guangzhou Women and Children’s Medical Center, Guangzhou Medical University, Guangzhou 510623, China; Guangzhou Women and Children’s Medical Center, Guangzhou Medical University, Guangzhou 510623, China; Guangzhou Women and Children’s Medical Center, Guangzhou Medical University, Guangzhou 510623, China; Guangzhou Women and Children’s Medical Center, Guangzhou Medical University, Guangzhou 510623, China; Guangzhou Women and Children’s Medical Center, Guangzhou Medical University, Guangzhou 510623, China; Center for Reproductive Medicine, Guangzhou Women and Children’s Medical Center, Guangzhou Medical University, Guangzhou 510623, China; China-Japan Union Hospital of Jilin University, Jilin University, Changchun 130033, China; Guangzhou Women and Children’s Medical Center, Guangzhou Medical University, Guangzhou 510623, China; Guangzhou Women and Children’s Medical Center, Guangzhou Medical University, Guangzhou 510623, China; Institute of Biophysics, Chinese Academy of Sciences, Beijing 100101, China

**Keywords:** Single-cell RNA sequencing, Full-length, Strand-specific, Antisense transcript, Mouse early embryonic development

## Abstract

Single-cell RNA sequencing (scRNA-seq) has transformed our understanding of cellular diversity with unprecedented resolution. However, many current methods are limited in capturing full-length transcripts and discerning strand orientation. Here, we present RAG-seq, an innovative strand-specific total RNA sequencing technique that combines not-so-random (NSR) primers with Tn5 transposase-mediated tagmentation. RAG-seq overcomes previous limitations by delivering comprehensive transcript coverage and maintaining strand orientation, which are essential for accurate quantification of overlapping genes and detection of antisense transcripts. Through optimized reverse transcription with oligo-dT primers, rRNA depletion via Depletion of Abundant Sequences by Hybridization (DASH), and linear amplification, RAG-seq enhances sensitivity and reproducibility, especially for low-input samples and single cells. Application to mouse oocytes and early embryos highlights RAG-seq’s superior performance in identifying stage-specific antisense transcripts, shedding light on their regulatory roles during early development. This advancement represents a significant leap in transcriptome analysis within complex biological contexts.

## Introduction

Single-cell RNA sequencing (scRNA-seq) technology has become an indispensable tool for unraveling cellular complexity and heterogeneity, a feat unattainable with traditional bulk RNA sequencing (RNA-seq) [1–4]. Since the introduction of the first single-cell sequencing technology by Tang et al. in 2009 [5], numerous methodologies have been developed to address diverse experimental requirements and biological questions. However, many contemporary scRNA-seq methods still exhibit limitations that hinder their broader application [6].

Many scRNA-seq methods employ oligo-dT primers to capture RNA and initiate complementary DNA (cDNA) synthesis following the lysis of individual cells. While this strategy can reduce ribosomal RNA (rRNA) contamination, it still presents several drawbacks. First, these methods are limited to detecting polyadenylated [poly(A)+] RNA, and are incapable of capturing non-polyadenylated [poly(A)−] RNA, which represents a substantial fraction of total cellular RNA. Second, the capture efficiency of oligo-dT primers is relatively low, which is estimated ranging between 7.1% and 15% in current protocols [4,7]. Furthermore, certain oligo-dT-based techniques display significant bias toward the 3′ ends or 5′ ends of RNA, such as CEL-seq/CEL-seq2 [2,8], Drop-seq [9], and STRT-seq [10].

Full-length RNA-seq enables comprehensive transcriptome analysis, facilitating the identification and characterization of splice variants, single-nucleotide polymorphisms (SNPs), and mutations. Among the methods designed for this purpose, Smart-seq2 [3] is a widely used scRNA-seq technique that combines an anchored oligo-dT primer with the template-switching capability of Moloney murine leukemia virus (M-MLV) reverse transcriptase. This combination allows for the full-length amplification of the transcripts [3]. Additionally, Tn5 transposase was found to bind RNA/DNA hybrids and target both DNA and RNA strands, transposing adapters to each strand, similar to its activity with double-stranded DNA (dsDNA). Building on this discovery, SHERRY [11] was developed as a rapid scRNA-seq method that provides near-full-length transcript coverage without the need for pre-amplification. Furthermore, SHERRY2 advances this approach by incorporating a highly efficient reverse transcriptase and leveraging the direct tagmentation capability of Tn5 transposase for RNA/DNA heteroduplexes, enabling the preparation of a more uniform full-length transcript library [12]. Both Smar-seq2 and SHERRY2 use a strategy of synthesizing full-length cDNA with oligo-dT primers, thereby biased to poly(A)+ RNAs. However, the current limitations of the two well-performing and widely used methods are unable to detect poly(A)− RNAs, which also lack strand specificity.

To address these challenges, two main approaches have been applied. Smart-seq-total [13] and VASA-seq [14] utilize dA-tailing of all RNA molecules, which allows for the capture of both poly(A)+ and poly(A)− RNA through oligo-dT primers. Additionally, some methods often employ random hexamer primers during reverse transcription (RT), which can capture full-length total cellular RNA [15–18]. Nonetheless, both approaches have inherent limitations, notably the conversion of rRNA, which constitutes up to 95% of total RNA [19,20], into cDNA. To address this issue, several strategies have been employed to remove rRNA sequences, including RNase H-mediated degradation [21], commercial rRNA depletion kits such as the Ribo-zero Gold rRNA Removal Kit (Illumina) [22], rRNA blocking probes (rRNA blockers) [18], and Depletion of Abundant Sequences by Hybridization (DASH) [17,23–25]. Additionally, not-so-random (NSR) primers have been utilized to prevent cDNA synthesis from rRNAs in RamDA-seq [26] and NSR [27].

Strand-orientation information is essential not only for accurately quantifying the expression of overlapping genes that are transcribed from both the plus and minus strands within the same genomic locus, but also for identifying unannotated antisense transcripts [28–32]. Genome-wide antisense transcription has been documented in various animal and plant species, including humans and mice [33]. Additionally, numerous studies suggest that antisense transcripts utilize diverse transcriptional and post-transcriptional regulatory mechanisms to perform a range of biological functions, including transcriptional interference, RNA masking, double-stranded RNA (dsRNA)-dependent mechanisms, and chromatin remodeling [34–36].

To date, three strand-specific, full-length total RNA-seq methods for single cells have been developed: Holo-seq [37], SMARTer [15], and VASA-seq [14]. Although Holo-seq provides complete strand specificity in profiling total RNA, it generates libraries with a high proportion of rRNA-mapped reads (50%−60%). SMARTer and VASA-seq have demonstrated excellent performance; however, they are limited to microplate or microfluidic platforms with bulk cells and involve complex procedures [14,15]. For certain samples, such as oocytes and early embryos, obtaining a sufficient number of cells is challenging, rendering strand-specific transcriptome analysis infeasible with current methods. Consequently, there remains a need for the development of novel, strand-specific, full-length total RNA sequencing techniques suitable for low-input samples or single cells.

In this study, we developed an NSR-primed and transposase tagmentation-mediated strand-specific total RNA sequencing technique, named RAG-seq. RAG-seq effectively integrates NSR primers with the DASH method, significantly reducing rRNA sequences in the libraries. Additionally, by leveraging the ability of Tn5 transposase to cut and tag RNA/cDNA hybrids directly, RAG-seq simplifies the experimental workflow by eliminating the need for separate fragmentation and adapter ligation steps.

RAG-seq demonstrates high sensitivity, reproducibility, and comprehensive full-length transcript coverage. Crucially, it preserves strand-orientation information, which facilitates accurate quantification of overlapping genes transcribed from opposite strands within the same genomic locus and aids in the identification of novel antisense transcripts. Furthermore, RAG-seq is not limited to a small number of cells; it is also effective with low-input purified total RNA and single mouse embryos.

Furthermore, we applied RAG-seq to analyze the transcriptomes of mouse oocytes and early embryos across various developmental stages. Our findings indicate that RAG-seq exhibits high sensitivity and reproducibility comparable to Smart-seq3 for gene expression profiling. Notably, RAG-seq demonstrates superior sensitivity in detecting antisense transcripts compared to Smart-seq3. RAG-seq identified a substantial number of antisense transcripts across different stages of early mouse embryonic development, with the number detected being at least eight times greater than that observed with Smart-seq3, except at the metaphase II (MII) stage. These antisense transcripts, similar to sense transcripts, exhibited distinct stage-specific expression patterns, regardless of whether they originated from protein-coding or non-coding genes. These results suggest that RAG-seq provides valuable insights into the potential regulatory roles of antisense transcripts during early embryonic development.

## Results

### Design of RAG-seq

**Figure 1:**
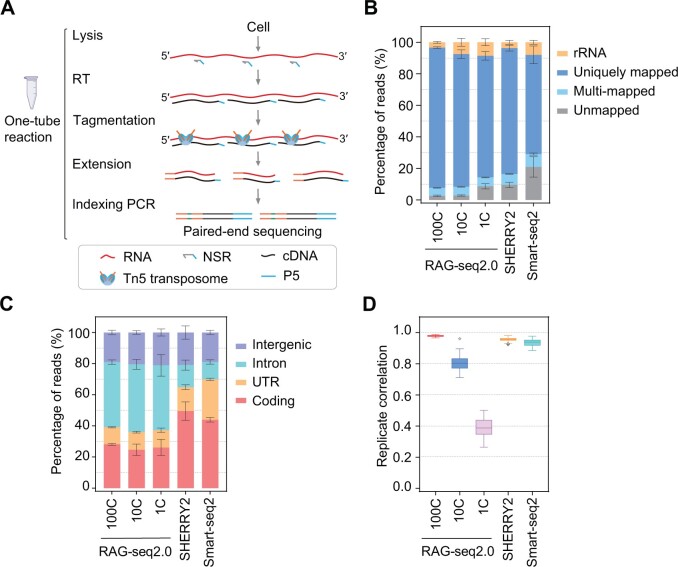
The workflow of RAG-seq1.0 and performance of RAG-seq2.0 **A**. Workflow of RAG-seq1.0. All experimental procedures can be performed within a single PCR tube. RNA released from a few cells or a single cell is reverse transcribed into first-strand cDNA using NSR primers. The RNA/cDNA hybrid is then directly tagmented by Tn5 transposome, followed by gap-filling. Ultimately, paired-end sequencing libraries are prepared through indexing PCR. **B**. Mapping statistics illustrating the percentages of rRNA, uniquely mapped, multi-mapped, and unmapped reads in libraries generated using RAG-seq2.0, SHERRY2, and Smart-seq2 protocols in HEK293 cells. **C**. Distribution of uniquely mapped reads across genome features. The percentages of reads aligned to coding, UTR, intronic, and intergenic regions are shown. **D**. Box-and-whisker plot showing the pairwise correlation of gene expression within replicates for the three RNA-seq protocols. The center line represents the median, the box represents the first and third quartiles (Q1 and Q3), and the whiskers indicate the most extreme data points within 1.5× the interquartile range (from Q1 to Q3). In (B−D), for RAG-seq2.0, HEK293 cells were analyzed under the following conditions, 100C, 100 cells (*n* = 4); 10C, 10 cells (*n* = 12); 1C, a single cell (*n* = 7); SHERRY2 and Smart-seq2 published data from single HEK293 cells were downloaded from the NCBI SRA (PRJNA879104). In (B and C), data are presented as mean ± SD. PCR, polymerase chain reaction; RT, reverse transcription; NSR, not-so-random; cDNA, complementary DNA; rRNA, ribosomal RNA; P5, P5 adaptor; UTR, untranslated region; RNA-seq, RNA sequencing; NCBI, National Center for Biotechnology Information; SRA, Sequence Read Archive; SD, standard deviation.

The RAG-seq1.0 workflow is illustrated in **Figure 1**A. RNA released from cell lysate is first reverse transcribed into cDNA using the NSR primers. Subsequently, RNA/cDNA hybrids are directly tagmented by Tn5 transposase, which is pre-loaded with an adaptor containing a sequencing primer binding site. Leveraging the “cut and paste” capabilities of Tn5 transposase, a second adaptor is rapidly appended to the 3′ end of the cDNA without second-strand synthesis, dsDNA fragmentation, and adaptor ligation. Following gap-filling and strand extension, the cDNA library is amplified using index polymerase chain reaction (PCR) primers.

Importantly, RAG-seq1.0 preserves strand orientation information during sequencing, as the cDNA library is flanked by two distinct adaptor sequences that label the 5′ and 3′ ends of the original RNA molecules. This design ensures that strand information is maintained throughout the sequencing process. To enhance sensitivity and minimize PCR bias, especially for low-input samples or single cells, RAG-seq2.0 incorporates linear amplification technology. RNA/cDNA hybrid is fragmented by the Tn5 transposome, which consists of Tn5 transposase and a modified transposon with a T7 promoter sequence. After strand extension, cDNA is converted into dsDNA with a T7 promoter through pre-PCR amplification. This dsDNA is then subjected to *in vitro* transcription (IVT) to linearly amplify it into RNA, followed by RT and second-strand synthesis to complete the library preparation (Figure S1A and B). The IVT step is particularly crucial for preparing libraries from small numbers of cells (Figure S1C).

In contrast to the prevalent RNA-seq methods such as Smart-seq2 and SHERRY2, which employ oligo-dT primers to capture poly(A)+ messenger RNAs (mRNAs) and reverse transcribe them into cDNA, RAG-seq2.0 utilizes NSR primers for initiating the RT reaction. While previous studies have demonstrated that NSR primers can mitigate cDNA synthesis from rRNA [26,27,38], RAG-seq2.0 libraries still show 23.4% to 13.1% of total sequence reads mapping to rRNA (Figure S2A). This residual rRNA mapping is likely due to the misannealing of NSR primers during RT, particularly when using a highly efficient reverse transcriptase. To address this issue, we employ DASH, a technology based on the CRISPR-Cas9 system, to further remove rRNA sequences following the pre-amplification. Specific single-guide RNAs (sgRNAs) targeting abundant rRNA sequences in RAG-seq2.0 libraries generated by NSR priming were designed for DASH. Compared to the non-DASH control, DASH treatment significantly reduced the percentage of rRNA reads from 23.4% to 6.45% and from 13.1% to 4.45% at the levels of 100 and 10 HEK293 cells, respectively (Figure S2A). Additionally, the read coverage of the sgRNA-targeted regions spanning the 18S and 28S rRNA loci decreased markedly after DASH treatment (Figure S2B). Through DASH treatment, the percentage of uniquely mapped reads increased from 66.92% to 86.1% and from 71.83% to 87.2% at the levels of 100 and 10 HEK293 cells, respectively (Figure S2C). The correlation between libraries with and without DASH treatment remained high (Figure S2D), indicating that the gene expression profiles of the libraries treated with DASH are largely comparable to those without DASH treatment. Meanwhile, sequence alignment revealed that the set of sgRNAs we designed is suitable to mouse samples, as the sequences targeted by these sgRNAs are conserved between human and mouse (Figure S2E). Furthermore, DASH treatment significantly reduced the percentage of rRNA reads in mouse blastocyst libraries, from 26.29% to 6.39% (Figure S2F and G), confirming the efficiency of DASH-mediated rRNA depletion in mouse samples. Overall, these results suggest that DASH effectively reduces the proportion of rRNA, thereby freeing up more sequencing capacity for other RNA species and potentially lowering sequencing costs.

### Performance of RAG-seq2.0

In the initial step of the RNA-seq protocol, RNA molecules are captured and converted into cDNA, making a high-efficiency and complete RT reaction crucial for sensitivity. To identify a highly efficient reverse transcriptase, we designed three pairs of primers targeting the 5′ end, middle region, and 3′ end of mRNA for quantitative real-time PCR (qRT-PCR) analysis, and compared the performance of four reverse transcriptases (Figure S3A). Ultimately, Maxima H Minus (Maxima H) was chosen for RAG-seq2.0 due to its superior and consistent efficiency in cDNA synthesis compared to the other enzymes (Figure S3B).

Following adjustments to the RAG-seq2.0 experimental parameters, we applied RAG-seq2.0 to profile transcriptomes from various amounts of HEK293 cells. We observed that the percentage of reads uniquely mapped to the genome in the RAG-seq2.0 libraries (77.1%) was comparable to the SHERRY2 libraries (79.8%) and higher than the Smart-seq2 libraries (62.9%) in single cells. Additionally, uniquely mapped reads were higher for 100 and 10 HEK293 cells, reaching 88.9% and 84.1%, respectively (Figure 1B). The distribution of uniquely mapped reads across human genome features in RAG-seq2.0 differed markedly from SHERRY2 and Smart-seq2. RAG-seq2.0 detected a high proportion of intronic reads, whereas SHERRY2 and Smart-seq2 identified more reads aligned to exonic regions [including coding regions and untranslated regions (UTRs)] (Figure 1C). This discrepancy is attributed to the NSR primers used in RAG-seq2.0, which capture more nascent RNA, while SHERRY2 and Smart-seq2 use oligo-dT primers that predominantly capture mRNA.

To assess the sensitivity and reproducibility of RAG-seq2.0, we compared its performance across various input amounts with SHERRY2 and Smart-seq2. SHERRY2 and Smart-seq2 demonstrated higher sensitivity, detecting more genes in single cells compared to RAG-seq2.0. Sensitivity in RAG-seq2.0 positively correlated with input quantity, with 100-cell and 10-cell inputs detecting 12,276 and 9271 genes [transcripts per million (TPM) > 1], respectively, closely aligning with SHERRY2 and Smart-seq2 (Figure S4A). Next, we evaluated the number of genes detected in each RNA type across the three different methods. RAG-seq2.0 could identify a spectrum of coding and non-coding transcripts, including mRNAs, long non-coding RNAs (lncRNAs), small nuclear RNAs (snRNAs), and microRNAs (miRNAs), similar to SHERRY2 and Smart-seq2 (Figure S4B). However, all three methods have lower sensitivity for short non-coding RNA molecules due to the size selection of fragments during library preparation. Additionally, replicate correlation in RAG-seq2.0 improved with increased input, with a replicate correlation of 0.978 for 100-cell input compared to 0.956 for SHERRY2 (Figure 1D). However, RAG-seq2.0 did not match the accuracy of SHERRY2 and Smart-seq2; the gene expression correlation between RAG-seq2.0 and NEBNext libraries was only half that between SHERRY2 and NEBNext (Figure S4C). This discrepancy may be due to NEBNext, like SHERRY2 and Smart-seq2, detecting primarily poly(A)+ mRNA, whereas RAG-seq2.0 captures both poly(A)+ mRNA and poly(A)− RNA. Furthermore, RAG-seq2.0 exhibited near-complete gene body coverage, with slight unevenness toward the 3′ end compared to SHERRY2 and Smart-seq2 (Figure S4D). In summary, RAG-seq2.0 is a full-length total RNA-seq method that demonstrates high sensitivity and reproducibility with small cell numbers.

### Analysis of antisense transcripts by RAG-seq2.0

**Figure 2:**
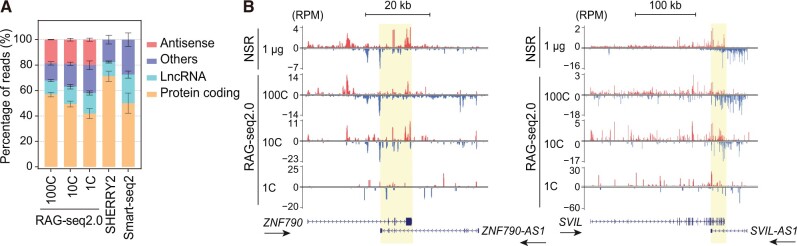
Antisense transcripts detected by RAG-seq2.0 **A**. Detection of various RNA biotypes using three RNA-seq methods. The percentage of total reads mapped to all annotated genes for each RNA biotype in the libraries constructed by RAG-seq2.0, SHERRY2, and Smart-seq2 protocols. Data are presented as mean ± SD. **B**. Tracks showing antisense transcripts *ZNF790-AS1* and *SVIL-AS1* detected at the *ZNF790* and *SVIL* genomic loci by RAG-seq2.0 and NSR. Sense and antisense reads are depicted in red and blue, respectively. The overlapping regions between sense and antisense transcripts are indicated by a yellow shadow. Arrows indicate the transcription direction (5′−3′) for each RefSeq gene. NSR represents strand-specific bulk RNA-seq data obtained using 1 μg of total RNA input. The figure was drawn using the UCSC gene browser. LncRNA, long non-coding RNA; RPM, reads per million mapped reads.

We assessed RAG-seq2.0’s capability to profile various RNA species, including its strand-specific analysis of both poly(A)+ and poly(A)− RNA. In RAG-seq2.0 data, 56.8%−44.6% of reads mapped to protein-coding genes, and 10.8%−5% mapped to lncRNAs. In contrast, SHERRY2 and Smart-seq2, which utilize poly(A) enrichment, showed higher read mapping to protein-coding genes and lncRNAs. Notably, RAG-seq2.0 uniquely detected antisense transcripts due to its strand-specific nature (**Figure 2**A). Antisense transcripts, many of which are unannotated, are crucial for regulating gene transcription, translation, and RNA degradation, potentially forming autoregulatory networks that modulate gene expression [34]. For instance, antisense transcripts such as *ZNF790-AS1*, *SVIL-AS1*, and *RNF213-AS1*, which were detected by bulk RNA-seq (NSR), were also identified by RAG-seq2.0 in both small- and single-cell analyses (Figure 2B, Figure S5). The strand-specific information provided by RAG-seq2.0 enables accurate read counting from overlapping genomic loci transcribed from opposite strands, such as *ENSG00000290058* within the *NUDT19* gene (Figure S5).

### Optimization of RAG-seq2.0 protocol

**Figure 3:**
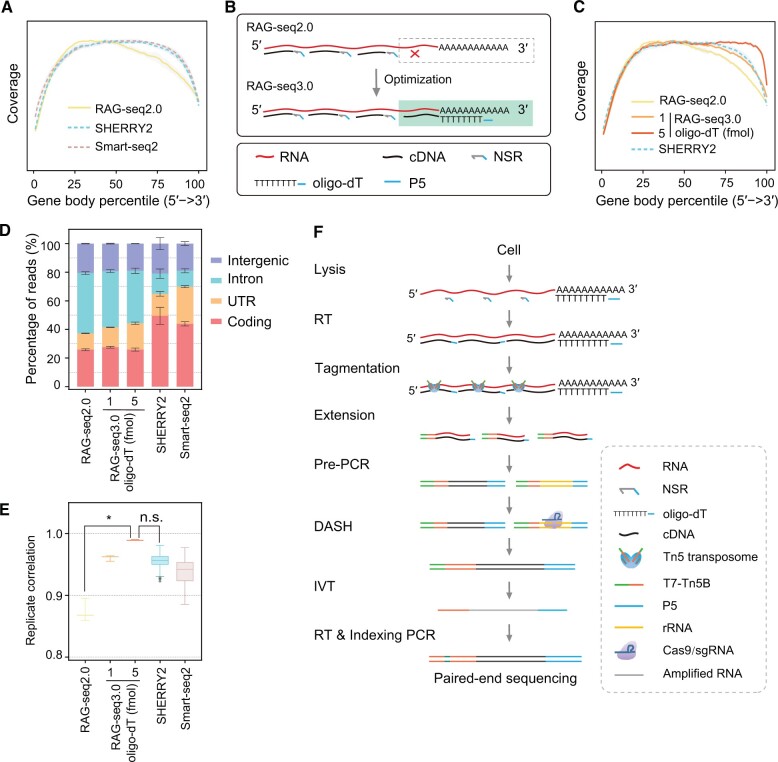
Optimization of RAG-seq2.0 protocol **A**. Comparison of read coverage across the gene body for RAG-seq2.0, SHERRY2, and Smart-seq2 methods. RAG-seq2.0 libraries were constructed using NSR primers alone. The gray region represents the SD of the normalized depth among replicates. **B**. Optimization of RT conditions in the RAG-seq3.0 protocol. The RAG-seq3.0 protocol employs both NSR and oligo-dT primers for RT. **C**. Comparison of read coverage across the gene body for the RAG-seq2.0, RAG-seq3.0, and SHERRY2. The gray region represents the SD of the normalized depth among replicates. **D**. Comparison of the distribution of uniquely mapped reads across genome features under different RT conditions. The percentages of reads aligned to coding, UTR, intronic, and intergenic regions are shown. Data are presented as mean ± SD. **E**. Box-and-whisker plot showing the pairwise correlation of gene expression within replicates for the RAG-seq2.0, RAG-seq3.0, SHERRY2, and Smart-seq2 protocols. The center line represents the median, the box represents the first and third quartiles (Q1 and Q3), and the whiskers indicate the most extreme data points within 1.5× the interquartile range (from Q1 to Q3). *P* values were determined by one-way ANOVA followed by Bonferroni’s multiple comparisons test (*, *P* < 0.05; n.s., not significant). **F**. Workflow of the RAG-seq3.0 protocol. The RAG-seq3.0 protocol was optimized through the utilization of three procedures: RT primed by NSR and oligo-dT primers, rRNA depletion with DASH after pre-amplification, and linear amplification of RNA by IVT. In (A and C−E), for RAG-seq2.0 and RAG-seq3.0, 10 HEK293 cells were used in (A) (*n* = 12) and (C−E) (*n* = 3); SHERRY2 and Smart-seq2 published data from single HEK293 cells were downloaded from the NCBI SRA (PRJNA879104). DASH, Depletion of Abundant Sequences by Hybridization; IVT, *in vitro* transcription.

Although RAG-seq2.0 provides full-length coverage, a slight unevenness toward the 3′ end was observed compared to SHERRY2 and Smart-seq2 (**Figure 3**A, Figure S4D). This bias may result from the incomplete RT of the RNA 3′ end, as NSR primers bind randomly to this region. In contrast, oligo-dT primers specifically bind to RNA poly(A) tails, enhancing RT efficiency at the 3′ end. To improve gene body coverage uniformity, we incorporated both NSR and oligo-dT primers into the RT reaction instead of using NSR primers alone (Figure 3B), naming the optimized protocol RAG-seq3.0.

To avoid introducing a 3′ end bias due to excessive oligo-dT primers, we performed a titration to determine the optimal concentration of oligo-dT primer for RAG-seq3.0. Coverage comparisons between RAG-seq2.0, RAG-seq3.0, and two published methods (SHERRY2 and Smart-seq2) revealed that gene body coverage became more uniform with increasing amounts of oligo-dT primer (Figure S6A). Using 1 fmol of oligo-dT primer rendered RAG-seq3.0 coverage comparable to SHERRY2 and Smart-seq2. Increasing the oligo-dT primer amount to 5 fmol significantly enhanced coverage uniformity, particularly at the 3′ end, outperforming both SHERRY2 and Smart-seq2 (Figure 3C, Figure S6A). Additionally, the percentage of UTR reads increased with higher oligo-dT primer concentrations. When 5 fmol of oligo-dT primer was used, the percentage of UTR reads increased by 7% compared to the no oligo-dT primer condition (18.42% *vs.* 11.36%) (Figure 3D, Figure S6B). The sensitivity of RAG-seq3.0 also improved with 5 fmol of oligo-dT primer, detecting an average of 10,307 genes (TPM > 1), which is 826 more genes (8.71%) than those detected by RAG-seq2.0 (Figure S6C). Moreover, replicate correlation improved with increased oligo-dT primer amounts (Figure S6D). With 1 fmol of oligo-dT primer, RAG-seq3.0 achieved a correlation of 0.960, higher than RAG-seq2.0 (0.874). Increasing to 5 fmol of oligo-dT primer resulted in a further improved replicate correlation (0.989) (Figure 3E, Figure S6D), indicating high reproducibility of the optimized RAG-seq3.0 at the level of a small number of cells. The accuracy of the RAG-seq3.0 also improved at the 10-cell level, as evidenced by a higher correlation in gene expression with NEBNext libraries compared to that of RAG-seq2.0 (R = 0.639−0.726 *vs.* R = 0.566) (Figure S6E). In summary, incorporating 5 fmol of oligo-dT primer into the RT reaction enhanced the performance of the RAG-seq3.0 protocol relative to RAG-seq2.0.

Next, we assessed the performance of the RAG-seq3.0 protocol with low-input purified total RNA. Although the exon rate remained relatively stable, the number of detected genes using RAG-seq3.0 increased at both 1 ng and 10 ng input levels compared to RAG-seq2.0 (Figure S7A and B). Additionally, replicate correlation significantly improved, reaching 0.983 at 1 ng and 0.992 at 10 ng (Figure S7C). Furthermore, the gene expression correlation between RAG-seq3.0 and NEBNext libraries, generated using a conventional RNA-seq method, showed notable enhancement (Figure S7D). Importantly, the gene body coverage in RAG-seq3.0, particularly at the 3′ end of RNA molecules, demonstrated superior performance (Figure S7E).

A comprehensive RAG-seq3.0 protocol was established through iterative improvements, including the optimization of the RT primers for cDNA synthesis, rRNA depletion with DASH, and linear amplification through IVT (Figure 3F). The RAG-seq3.0 protocol exhibits enhanced sensitivity, reproducibility, and accuracy compared to its predecessor. These advancements suggest that RAG-seq3.0 is highly suitable for strand-specific transcriptome analysis of challenging samples, such as oocytes and early embryos.

### Transcriptome analysis of mouse early embryos using RAG-seq3.0

Antisense transcription is a widespread phenomenon across various species, including humans and mice, and plays critical roles in biological processes and diseases such as cancer, neurological disorders, and cardiovascular diseases [39–41]. Strand-specific RNA-seq is essential for understanding the expression and function of natural antisense transcripts. However, most RNA-seq methods used to profile early mouse embryo transcriptomes lack strand-orientation information, including Tang et al.’s method [5], SUPeR-seq [42], Smart-seq2 [43–46], and RamDA-seq [26,47]. Due to limitations in the detection capabilities of RNA-seq methods and sample availability, the study of antisense transcript expression and its regulatory roles in early mouse embryos remains insufficient.

**Figure 4:**
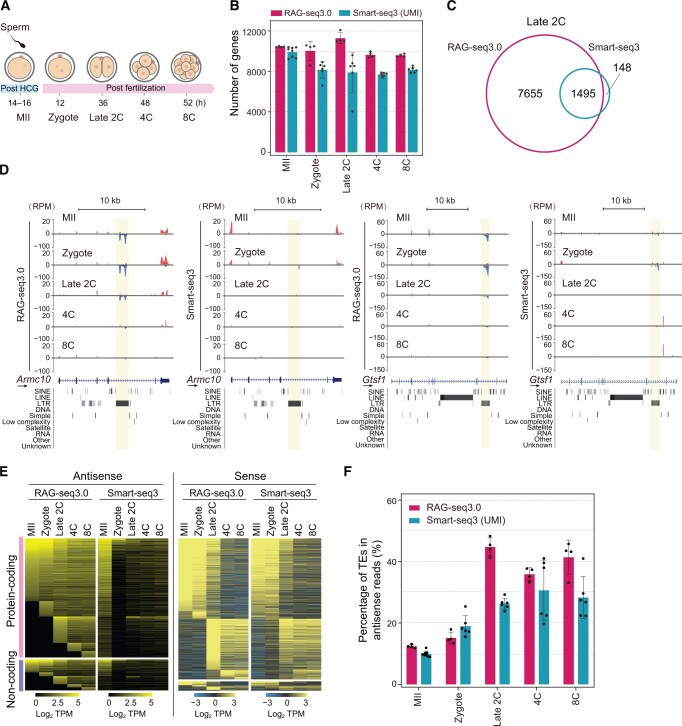
Transcriptome analysis of mouse early embryos using RAG-seq3.0 **A**. Schematic depicting the collection of mouse early embryos for RAG-seq3.0 and Smart-seq3 analysis. Samples including MII oocytes, zygotes, late 2-cell (late 2C) embryos, 4-cell (4C) embryos, and 8-cell (8C) embryos were analyzed. **B**. Comparison between the number of genes (TPM > 1) detected at each developmental stage by RAG-seq3.0 and Smart-seq3. **C**. Venn diagram showing the overlap of antisense transcripts detected by RAG-seq3.0 and Smart-seq3 at the late 2C stage. **D**. Tracks showing antisense transcripts detected in the *Armc10* and *Gtsf1* genomic loci by RAG-seq3.0 and Smart-seq3. Sense and antisense reads are shown in red and blue, respectively. Antisense transcripts in genomic regions are marked in shadow. Arrows indicate the transcription direction (5′−3′) for each RefSeq gene. **E**. Heatmaps showing the stage-specific expression (TPM) patterns of antisense and sense transcripts detected by RAG-seq3.0 and Smart-seq3. **F**. Percentage of reads mapped to TEs among total antisense transcript reads. In (B) and (F), data are presented as mean ± SD. For RAG-seq3.0, *n* = 4 per stage; for Smart-seq3, *n* = 8 or 6 per stage. Strand-specific 5′ UMI-containing reads from Smart-seq3 were used for analysis. HCG, human chorionic gonadotropin; MII, metaphase II; SINE, short interspersed nuclear element; LINE, long interspersed nuclear element; LTR, long terminal repeat; UMI, unique molecular identifier; TPM, transcript per kilobase per million mapped reads; TE, transposable element.

To address these limitations, we applied the RAG-seq3.0 protocol to early mouse embryos and concurrently performed Smart-seq3, which provides strand-specific 5′ unique molecular identifier (UMI) reads and non-strand-specific internal reads [48]. Our analysis covered MII oocytes, zygotes, late two-cell (late 2C) embryos, four-cell (4C) embryos, and eight-cell (8C) embryos (**Figure 4**A). The performance of RAG-seq3.0 was comparable to Smart-seq3, with similar distributions of uniquely mapped reads across genome features at different developmental stages. Specifically, higher proportions of reads mapped to exonic regions (including coding regions and UTRs) in MII oocytes and zygotes, while intergenic reads increased in late 2C embryos and subsequently decreased in 4C and 8C stages (Figure S8A). This pattern may be linked to the high expression of *de novo* non-coding RNAs during zygotic genome activation (ZGA) [49].

We evaluated RAG-seq3.0 sensitivity by counting the number of detected genes at each developmental stage. MII oocytes, zygotes, and late 2C embryos had higher gene detection counts on average (10,432, 10,015, and 11,284 genes, respectively), with a slight reduction in 4C and 8C stages, consistent with Smart-seq3 findings (Figure S8B). This reduction likely reflects maternal RNA degradation during embryonic development. Notably, RAG-seq3.0 detected more genes than Smart-seq3’s 5′ UMI reads, except at the MII stage (Figure 4B). Reproducibility was assessed by calculating correlations in gene expression between individual embryos, with average correlations exceeding 0.9, similar to Smart-seq3, indicating high reproducibility of RAG-seq3.0 (Figure S8C). Both methods provided full-length coverage of gene bodies, facilitating a comprehensive analysis of full-length transcripts (Figure S8D). Principal component analysis (PCA) of gene expression data from embryos at various developmental stages revealed distinct clustering, with a notable shift at the late 2C stage, underscoring significant changes in transcriptome expression that are critical for early embryonic development (Figure S8E).

Antisense transcripts, many of which are annotated, are vital regulators of gene transcription, translation, and RNA degradation, and they play significant roles in processes such as differentiation, development, and disease. In our study, we profiled antisense transcripts across various development stages using RAG-seq3.0. RAG-seq3.0 detected a substantially greater number of antisense transcripts compared to Smart-seq3, with the exception of the MII stage (Figure S9A). At the late 2C stage, RAG-seq3.0 identified 90% of the antisense transcripts detected by Smart-seq3, demonstrating its superior sensitivity for capturing antisense transcription events (Figure 4C, Figure S9A). The expression of antisense transcripts varied with the developmental stages, with some transcripts detected exclusively by RAG-seq3.0 and others by both RAG-seq3.0 and Smart-seq3 (Figure 4D, Figure S9B).

Moreover, RAG-seq3.0 revealed pronounced stage-specific expression patterns for antisense transcripts, regardless of whether they were associated with protein-coding or non-coding genes, whereas Smart-seq3 did not reveal such distinct patterns (Figure 4E, left). In contrast, the expression pattern of the sense transcripts was similar between the two methods, with a notable increase in gene expression observed at the late 2C stage (Figure 4E, right), coinciding with ZGA. Additionally, we investigated the overlap between antisense transcripts and repetitive elements (Figure 4F).

## Discussion

In this study, we introduce RAG-seq, a cutting-edge strand-specific total RNA-seq method based on NSR priming and Tn5 transposase tagmentation. Through rigorous optimization and validation, RAG-seq has demonstrated outstanding sensitivity, reproducibility, and comprehensive full-length transcript coverage. Crucially, RAG-seq retains strand orientation information, enabling precise identification of antisense transcripts, which is a significant advancement over traditional RNA-seq methods. SHERRY and SHERRY2 have previously utilized the Tn5 transposome for rapid and cost-effective RNA-seq by tagmenting RNA/cDNA hybrids [11,12]. However, these methods lose strand orientation information due to the transposition of adaptors at both the 5′ and 3′ ends of the RNA/cDNA hybrid. RAG-seq overcomes this limitation by preserving strand specificity through NSR priming and tagmentation with Tn5 transposase carrying a single adaptor (Figure 1A, Figure 3F).

Despite its strengths, initial RAG-seq2.0 libraries exhibited a notable proportion (23.4%−13.1%) of rRNA sequences when only NSR primers were employed. (Figure S2A). To address this, we incorporated an rRNA depletion step using DASH after pre-amplification [17,23–25]. Through DASH-mediated rRNA depletion, the percentage of rRNA reads was significantly reduced to 3.37%−8.6%, resulting in uniquely mapped reads as high as 77.1% − 88.9%, which is higher than that in Smart-seq2 and SHERRY2 (Figures 1B, Figure S2A and C). Furthermore, although RAG-seq2.0 provided full-length transcript coverage, a minor bias toward the 3′ end persisted compared to SHERRY2 and Smart-seq2 (Figure 3A, Figure S4D). This bias is likely due to incomplete RT at the 3′ end caused by the random hybridization of NSR primers. The integration of oligo-dT primers, which specifically bind to poly(A) tails, significantly enhanced 3′ end coverage and overall transcript coverage, thereby improving the sensitivity, reproducibility, and accuracy of RAG-seq3.0, particularly for low-input samples (Figure 3, Figures S6 and S7).

Antisense transcripts, which are extensively expressed in mammals and plants, play crucial roles in regulating the expression of their corresponding sense genes at various levels, including transcriptional, co-transcriptional, or translational. While extensive research on antisense-mediated gene regulation has been conducted in yeast [50], studies in mammals, especially during early embryonic development, remain sparse. Traditional bulk RNA-seq methods face challenges related to sample size and strand orientation, while the majority of scRNA-seq methods lose strand information during library preparation. Although several strand-specific scRNA-seq methods, such as SMARTer [15], VASA-seq [14], and scComplete-seq [51], have demonstrated excellent performance, these methods are designed based on high-throughput platforms and are therefore unsuitable for early mouse embryo research. RAG-seq addresses these limitations by offering strand-specific transcriptome profiling of early mouse embryos, an advancement not achieved by prevalent scRNA-seq methods such as Tang et al.’s method [5], Smart-seq2 [3], Smart-seq-total [13], and RamDA-seq [26,47]. RAG-seq’s ability to identify antisense transcripts in early mouse embryos provides valuable insights into their roles during early development (Figure 4C, Figure S9A).

Despite its distinctive advantages, RAG-seq has potential areas for further improvement. Barcoding technology, which is commonly used in RNA-seq methods, could enhance sample throughput, reduce costs, and shorten processing time [15,52]. Currently, the use of NSR primers restricts the pooling of samples in the early stages of experimental processing without barcodes, limiting high-throughput multiplex analysis. Additionally, while RAG-seq exhibits comparable performance to Smart-seq3 in early mouse embryos (Figure 4, Figure S8), further optimization is needed to enhance its performance with single HEK293 cells, a commonly studied mammalian cell line.

In summary, RAG-seq represents a significant advancement in transcriptome analysis, particularly for rare embryos at the early stages of mammalian development. Its capacity for comprehensive strand-specific coverage offers a powerful tool for understanding the transcriptomic landscape during mammalian development, providing new insights into gene regulation and expression.

## Materials and methods

### Cell culture

HEK293 cells (ATCC) were cultured in Dulbecco’s modified Eagle medium (DMEM; Catalog No. 11965092, Gibco, Carlsbad, CA) supplemented with 10% fetal bovine serum (FBS; Catalog No. 1600044, Gibco) and 1% penicillin−streptomycin (Catalog No. 15140122, Gibco) at 37°C with 5% CO_2_. Adherent cells were washed twice with phosphate buffer saline (PBS) and dissociated into single-cell suspension with 0.05% Trypsin-EDTA (Catalog No. 25300062, Gibco) at 37°C for 3 min. The Trypsin-EDTA was then inactivated with double volume of culture medium. Cells were collected by centrifugation at 200 *g* for 5 min and resuspended for downstream experiment or subculture.

### Cell isolation and lysis

HEK293T cells were digested and washed twice with ice-cold PBS (Catalog No. 10010023, Gibco), and then diluted in PBS containing 1% bovine serum albumin (BSA). Cells were selected by mouth pipetted under a microscope and transferred into 0.2-ml PCR tube containing 2 μl cell lysis buffer, which was composed of 0.5% Triton X-100 (Catalog No. 85111, Thermo Fisher Scientific, Waltham, MA), 4 U RiboLock RNase inhibitor (Catalog No. EO0382, Thermo Fisher Scientific), 1× DNase buffer (Catalog No. 18068015, Thermo Fisher Scientific), and 0.4 U DNase I Amplification Grade (Catalog No. 18068015, Thermo Fisher Scientific). The selected cells were then lysed and incubated at 20°C for 30 min to digest the genomic DNA. After processing, the cell lysate solution was used for next experimental step or stored at −80°C until used.

### IVF and mouse early embryo collection

C57BL/6 and PWD/PhJ mice were purchased from Charles River (Beijing, China). All mice were maintained under constant humidity and temperature in specific pathogen-free (SPF) facilities at the Laboratory Animal Resources, Chinese Academy of Sciences, with free access to food and water.

Adult female C57BL/6 mice (6−8 weeks old) and adult male PWD/PhJ mice (8−10 weeks old) were used as oocyte and sperm donors, respectively, for IVF. C57BL/6 female mice were intraperitoneally injected with pregnant mare serum gonadotropin (PMSG, 5 IU), followed by human chorionic gonadotropin (HCG, 5 IU) 48 h later. MII oocytes were isolated and collected from the ampullary region of the oviduct of superovulated female mice 14−16 h after HCG injection for IVF and RNA-seq. Sperm obtained from PWD/PhJ males were capacitated for 1 h in HTF medium (Catalog No. MR-070-D, Sigma-Aldrich, St. Louis, MO) at 37°C with 5% CO_2_. For IVF, oocytes were incubated with the capacitated sperm in HTF medium at 37°C with 5% CO_2_ for 5−6 h, then washed and cultured in KSOM medium (Catalog No. MR-106, Sigma-Aldrich) to reach the corresponding stage. Embryos were collected at the following time points post IVF: 12 h (zygote), 36 h (late 2-cell), 48 h (4-cell), and 52 h (8-cell).

### qRT- PCR

Total RNA was extracted from HEK293 cells using TRIzol reagent (Catalog No. 15596026, Thermo Fisher Scientific) according to the manufacturer’s instructions and treated with the TURBO DNase (Catalog No. AM2238, Thermo Fisher Scientific). cDNA was synthesized at 25°C for 5 min, 37°C for 15 min, 40°C for 90 min, 70°C for 15 min, and held on at 4°C. qRT-PCR was performed using the Hieff qPCR SYBR Green Master Mix (Catalog No. 11201ES03, Yeasen, Shanghai, China) on the CFX Connect Real-Time PCR Detection System (Hercules Bio-Rad, CA). All primers used for qRT-PCR are listed in Table S1. Data analysis was performed using Bio-Rad CFX Manager Software v3.1 (Bio-Rad).

### Tn5 transposome assembly

Mosaic-end (ME) double-stranded adaptor T7-Tn5B was obtained by annealing 100 μM T7-Tn5-MEB oligonucleotides with equimolar Tn5-ME-rev oligonucleotides in annealing buffer (10 mM Tris-HCl pH 7.5, 10 mM NaCl). Samples were incubated for 5 min at 94°C and then cooled down slowly (1°C/min) to 10°C. Tn5 transposomes were then assembled as described previously [53]. The assembled Tn5 transposomes were stored at −20°C until use. The oligos used are listed in Table S2.

### RAG-seq method

#### RT

First, 1 μl of 0.12 U/μl thermolabile Proteinase K (Catalog No. P8111S, New England Biolabs, Beverly, MA) was added to the cell lysate, incubated at 25°C for 30 min to remove DNase I, and then heat-inactivated at 55°C for 10 min. Subsequently, 50 pmol NSR primers, 5 fmol P5-oligo-dT primer, 0.8 μl 25 mM dNTP (Catalog No. A610057, Sangon Biotech, Shanghai, China), and 0.2 μl nuclease-free water were added to the 3 μl cell lysate sample. Then the sample was incubated at 72°C for 3 min and immediately placed on ice to denature the RNA. Next, 5 μl RT mixture [2 μl 5× RT buffer, 100 U Maxima H Minus Reverse Transcriptase (Catalog No. EP0751, Thermo Fisher Scientific), 10 U RiboLock RNase inhibitor, 2 μl 5 M Betaine (Catalog No. B0300, Sigma-Aldrich), and 0.25 μl nuclease-free water] was added to the sample tube, and incubated at 25°C for 5 min, 37°C for 15 min, 40°C for 90 min, 70°C for 15 min, and held on at 4°C.

#### Tagmentation and preamplification

A total of 10 μl tagmentation mixture containing 1 μl Tn5 transposome, 4 μl 5× TD buffer [50 mM Tris-HCl pH7.5 (Catalog No. 15567027, Thermo Fisher Scientific), 25 mM MgCl_2_ (Catalog No. 63069, Sigma-Aldrich), 50% DMF (Catalog No. D4551, Sigma-Aldrich), 4.25 mM ATP (Catalog No. P0756S, New England Biolabs)], 4.5 μl 40% PEG8000 (Catalog No. 89510, Sigma-Aldrich), and 0.5 μl nuclease-free water was added to the RT product. The reaction mixture was incubated at 55°C for 30 min, followed by adding 1 μl 0.4% SDS to inactivate the transposase at 55°C for 7 min. Gap filling and strand extension were performed by adding 1 μl of 8 U/μl Bst3.0 DNA polymerase (Catalog No. M0374M, New England Biolabs) and 1× Q5 high-fidelity master mix (Catalog No. M0494L, New England Biolabs) to the tagmentation product. The mixture was incubated at 72°C for 15 min and then terminated at 80°C for 5 min. Finally, 3 μl 2× Q5 high-fidelity master mix, 4.5 pmol T7 custom B, and 4.5 pmol custom P5 primer were used to perform PCR preamplification. PCR amplification was carried out using the following program: 94°C for 2 min; 2 cycles of 94°C for 10 s, 40°C for 2 min, 72°C for 1 min; 2 cycles of 94°C for 10 s, 60°C for 30 s, 72°C for 1 min; *n* cycles of 94°C for 10 s, 60°C for 30 s, 72°C for 1 min with an additional 10 s added at each cycle; 72°C for 5 min, held on at 12°C. The PCR cycles “*n*” depends on the amount of the input sample (*n* = 11 for single cell, *n* = 8 for 10 cells and 1 ng RNA, *n* = 5 for 100 cells and 10 ng RNA).

#### rRNA depletion

rRNA fragments were removed by DASH as described in previous studies [17,23–25]. Briefly, sgRNAs targeting 18S and 28S rRNAs (Table S3) were designed and prepared by IVT using T7 RNA polymerase. The sgRNAs were then purified, pooled in equal amounts, aliquoted, and stored at −80°C. Cas9/sgRNA complexes were assembled by mixing Cas9 nuclease and sgRNA at a 1:2 molar ratio in 1× buffer 3.1 (Catalog No. B9000, New England Biolabs), followed by preincubation at 37°C for 15 min. Subsequently, 5 μl complex mixture was incubated with 50 μl PCR products at 37°C for 2 h. Following digestion, Cas9 nuclease was inactivated with 1 μl of Thermolabile Proteinase K at 37°C for 15 min, and then heated at 55°C for 10 min to terminate the reaction. The sample was purified using 1× AMPure XP beads according to the manufacturer’s instructions (Catalog No. A63881, Beckman Coulter, Miami, FL). Finally, 5 μl nuclease-free water was added to the tube to resuspend beads and elute dsDNA off beads (Note: do not discard the beads).

#### IVT and RNA purification

A total of 5 μl of IVT reaction mixture [1 μl 10× reaction buffer, 2 μl 10 mM NTP (Catalog No. B600056, Sangon Biotech), 0.5 μl RNase inhibitor, 0.5 μl 0.1 U/μl YIPP (Catalog No. M2403, New England Biolabs), and 1 μl 50 U/μl T7 RNA polymerase (Catalog No. EG201225S, YuGong Biotech, Lianyungang, China)] was added to the PCR tube and incubated at 37°C overnight for 10−16 h. After the reaction, 45 μl 10 mM Tris-HCl (pH 8.0) was added to the sample tube to bring the total volume to 55 μl. Subsequently, 55 μl of HXP Buffer (20% PEG 8000, 2.5 M NaCl) was added to the IVT solution to clean up the amplified RNA. Finally, the RNA was eluted in 11 μl nuclease-free water.

#### Second RT, PCR amplification, and sequencing

The linear amplified RNA was then mixed with 1 μl 10 mM dNTPs (Catalog No. A610056, Sangon Biotech) and 1 μl 10 μM P5 primer. The mixture was incubated at 65°C for 5 min and immediately placed on ice for at least 2 min. Next, 4 μl 5× RT buffer, 1 μl RNase inhibitor, 1 μl 200 U/μl SuperScript III (Catalog No. 18080044, Thermo Fisher Scientific), and 1 μl nuclease-free water were added to the sample. The mixture reaction was incubated at 25°C for 5 min, 50°C for 1 h, followed by 70°C for 15 min, and then hold at 4°C. Next, 10 μl cDNA was used for the final PCR amplification to prepare the library. The cDNA was mixed with 0.5 μl of 10 μM P7 index primer, 0.5 μl of 10 μM P5 primer, 25 μl of 2× Q5 high-fidelity master mix, and 14 μl nuclease-free water. PCR amplification was performed using the following program: 98°C for 2 min; 3−5 cycles of 98°C for 30 s, 60°C for 20 s, and 72°C for 2 min; 72°C for 5 min, then held at 12°C. The PCR product was purified with 0.8× AMPure XP SPRI beads and eluted in 20 μl TE buffer. The RAG-seq3.0 libraries were then quantified with a Qubit 3.0 Fluorometer (Life Technologies, Gaithersburg, MD) and sequenced on an Illumina NovaSeq6000 platform. The primers and oligos used are listed in Table S2.

### Bulk RNA-seq

For NSR RNA-seq, 1 μg of total RNA purified from HEK293 cells was used to prepare libraries following the NSR methods described in a previous study [27]. The primers and oligos used are listed in Table S2.

### Sequencing library preparation for Smart-seq3

Smart-seq3 library preparations were performed as previously described [48] with some modifications. Mouse embryos were collected and lysed in 0.2-ml PCR tube with 3 μl lysis buffer (4 U RNase inhibitor, 0.2% Triton X-100, 0.5 mM dNTPs, 1 μl of 10 μM Smart-seq3 oligo-dT primer). The tube was incubated at 72°C for 3 min to denature RNA. Next, 7 μl of RT mixture, containing 2 μl of 5× RT Buffer, 0.25 μl RNase inhibitor, 0.5 μl Maxima H Minus Reverse Transcriptase, 2 μl of 5 M Betaine, 0.12 μl of 500 mM MgCl_2_, 0.5 μl of 100 mM DTT, 0.5 μl of 10 μM TSO, and 1.15 μl nuclease-free water. RT and template switching were carried out at 42°C for 90 min followed by 10 cycles of 50°C for 2 min and 42°C for 2 min. The reaction was terminated by incubating at 85°C for 5 min. PCR pre-amplification was performed by adding 15 μl PCR mixture [12.5 μl of 2× KAPA HiFi ReadyMix (Catalog No. KK5603, Roche, Basel, Switzerland), 0.1 μl of 10 μM Smart-seq3 forward PCR primer, and 0.1 μl of 10 μM Smart-seq3 reverse PCR primer] to the RT product. PCR program was as follows: 98°C for 3 min; 15 cycles of 98°C for 20 s, 65°C for 20 s, and 72°C for 6 min; 72°C for 5 min. Subsequently, the PCR products were purified with 0.6× Ampure XP beads. Then 5 ng cDNA was used for the tagmentation reaction to prepare libraries. These libraries were sequenced on an Illumina NovaSeq 6000 platform. The primers and oligos used are listed in Table S2.

### RNA-seq data analysis

The published sequence data (SHERRY2 and Smart-seq2) used in this study can be accessed from the National Center for Biotechnology Information (NCBI) Sequence Read Archive (SRA: PRJNA879104) [12].

Adaptors and poly(A/T) sequences were trimmed, and bases with quality less than 20 and length of reads shorter than 20 bases were removed from the raw paired-end sequencing data by Cutadapt (v4.1) [54]. Trimmed reads were aligned to either the human genome (hg19) or the mouse genome (mm10), including the ribosomal DNA sequences (45S), using the STAR aligner (v2.4) [55]. Only unique alignment reads were utilized for the downstream analysis. Reads aligned to annotated gene features were counted using featureCounts (v1.6.3) [56]. The TPM values for annotated genes were calculated by Cufflinks (v2.2.1) [57], and genes with TMP > 1 were considered to be detected. Coverage across the gene body was calculated by counting the reads that were aligned at each position of the RefSeq transcripts with RSeQC (v2.6.4) [58]. The coverage uniformity was defined as the integral area between the coverage curve and the x-axis normalized by 100. For visualization of read coverage, bigWig files created from bedGraph files using the program bedGraphToBigWig [59] were uploaded to the UCSC genome browser. All Pearson correlations were measured between log_2_ reads per million mapped reads (RPM) values. PCA was carried out using R software (v3.1), and ggplot2 was used to draw graphs.

### Statistical analyses

Data are presented as mean ± SD. Statistical analyses were carried out using GraphPad software. The two-tailed unpaired *t*-test was used to compare the differences between two experimental groups, and one-way analysis of variance (ANOVA) followed by Bonferroni’s multiple comparisons was used to assess differences among multiple experimental groups. *P* values are indicated by asterisks in the figures as follows: *, *P* < 0.05; **, *P* < 0.01; ***, *P* < 0.001.

## Ethical statement

All animal breeding, housing, and experimental procedures were conducted in accordance with the guidelines of the Institutional Animal Care and Use Committee (IACUC) of Center for Animal Research, Institute of Biophysics, Chinese Academy of Sciences (Approval No. SYXK2020057).

## Supplementary Material

qzae072_Supplementary_Data

## Data Availability

The raw sequence data generated in the study have been deposited in the Genome Sequence Archive and Genome Sequence Archive for Human [60] at the National Genomics Data Center [61], Beijing Institute of Genomics, Chinese Academy of Sciences / China National Center for Bioinformation (GSA: CRA018566; GSA-Human: HRA008391), and are publicly accessible at https://ngdc.cncb.ac.cn/gsa/ and https://ngdc.cncb.ac.cn/gsa-human/.

## CRediT author statement

**Ping Xu:** Conceptualization, Investigation, Methodology, Resources, Validation, Visualization, Writing – original draft. **Zhiheng Yuan:** Resources, Investigation. **Xiaohua Lu:** Methodology, Resources. **Peng Zhou:** Resources, Validation. **Ding Qiu:** Conceptualization, Validation. **Zhenghao Qiao:** Software, Visualization. **Zhongcheng Zhou:** Writing – review & editing. **Li Guan:** Writing – review & editing. **Yongkang Jia:** Resources. **Xuan He:** Validation. **Ling Sun:** Resources, Writing – review & editing. **Youzhong Wan:** Conceptualization, Project administration, Supervision, Writing – review & editing. **Ming Wang:** Data curation, Formal analysis, Software, Visualization, Writing – original draft. **Yang Yu:** Conceptualization, Funding acquisition, Project administration, Supervision, Writing – review & editing. All authors have read and approved the final manuscript.
