# Supplementary material for: RAG-seq: NSR-primed and Transposase Tagmentation-mediated Strand-specific Total RNA Sequencing in Single Cells
Source: Genomics Proteomics Bioinformatics. 2024 Oct 10;22(5):qzae072. doi: 10.1093/gpbjnl/qzae072 (PMC11658833; doi:10.1093/gpbjnl/qzae072)
Supplement: qzae072_Supplementary_Data [file qzae072_supplementary_data.zip › supplementary material captions.docx]

**Supplementary material**

**Figure S1**  **The T7-Tn5 transposome scheme for RAG-seq2.0**

**A.** T7-Tn5 transposon and transposome. The T7-Tn5B transposon includes a 19 bp double-stranded transposase binding site and a T7 promoter sequence. The T7-Tn5B transposon and Tn5 transposase are mixed in equimolar amounts and dimerized to form the T7-Tn5 transposome. **B.** Diagram of the T7-Tn5 transposome work scheme. RNA/cDNA hybrid is fragmented and tagged by T7-Tn5B transposon. Following strand extension, the cDNA is converted to double-stranded DNA with a T7 promoter by pre-PCR amplification. IVT is then performed to linearly amplify the dsDNA into RNAs. **C**. Gel images showing that the construction of the RAG-seq2.0 library relies on IVT amplification, using 10 HEK293 cells. M, marker; Rep, replicate; bp, base pair; IVT, *in vitro* transcription; cDNA complementary DNA.

**Figure S2**  **Validation of rRNA depletion in RAG-seq2.0 libraries**

**A.** rRNA depletion mediated by DASH in HEK293 cells. The percentage of rRNA reads in libraries prepared with or without DASH treatment. **B.** Comparison of sequence read coverage of the human 45S rRNA sequence in libraries with and without DASH treatment. The red arrowhead indicates the target region of the sgRNA in panels (B), (E), and (G). **C.** Mapping statistics illustrating the percentages of uniquely mapped, multi-mapped, and unmapped reads in libraries prepared with or without DASH treatment. **D.** Pairwise correlation of gene expression (log_2_ RPM) with and without DASH treatment. **E.** Schematic diagram of sequence alignment of mouse and human 45S rRNA. Gray highlights conserved rRNA sequences in human and mouse. **F.** rRNA depletion mediated by DASH in mouse blastocyst RAG-seq2.0 libraries. The percentage of reads mapped to rRNAs in libraries with and without DASH treatment. **G.** Comparison of sequence read coverage of the mouse 45S rRNA sequence in libraries with and without DASH treatment. Conditions include HEK239 cell: 100C, 100-cell; 10C, 10-cell. Data are presented as mean ± SD (n = 4~7). *P* values were calculated by two-tailed unpaired *t-*test (***, *P* ＜ 0.001). DASH, Depletion of Abundant Sequences by Hybridization; NR, non-coding RNA; RPM, reads per million mapped reads.

**Figure S3**  **The testing of reverse transcriptase for the RAG-seq2.0 protocol**

**A**. Design of qPCR primers for evaluating the efficiency of different reverse transcriptases. Three pairs of qPCR primers were designed to target the 5' end, middle region, and 3' end of mRNA. Four reverse transcriptases were compared; Superscript ^TM^ II (SSII), M-MLV GIII (GIII), Superscript ^TM^ IV (SSIV), and Maxima H Minus (Maxima H). **B**. Efficiency comparison of different reverse transcriptases. A total of 100 ng of purified HEK293 RNA was reverse transcribed by each of the four reverse transcriptases under the same conditions; Reverse transcription efficiency for the *KMT2C* (16.8 kb) transcript was analyzed by qPCR. All groups were normalized to SSII. Data are presented as mean ± SD from three independent experiments. *P* values were calculated by one-way ANOVA followed by Bonferroni’s multiple comparisons test (**, *P* ＜ 0.01; ***, *P* ＜ 0.001). mRNA, messenger RNA; F, forward primer; R, reverse primer; *KMT2C*, *lysine methyltransferase* *2C*; qPCR, quantitative real-time PCR; SD, standard deviation.

**Figure S4**  **Performance of RAG-seq2.0**

**A.** Number of genes (TPM > 1) detected with RAG-seq2.0, Smart-seq2, and SHERRY2 in HEK293 cells. **B.** The number of genes (TMP > 1) detected in each RNA type across the three different methods. **C.** Correlation of gene expression between small numbers of cells or single HEK293 cells and bulk RNA extracted from HEK293T cells (NEBNext). **D.** Read coverage across the gene body for the three methods. The gray area indicates the standard deviation of normalized depth across replicates. For RAG-seq2.0, HEK293 cells were analyzed under the following conditions: 100C, 100 cells (n = 4); 10C, 10 cells (n = 12); and 1C, a single cell (n = 7). Bulk RNA-seq (NEBNext) published data were downloaded from the NCBI SRA (PRJNA879104). Data in panels (A) and (B) are presented as mean ± SD. For box and whiskers plots in panel (C), the center line represents the median, the upper and lower lines of the box represent the first and third quartiles (Q1 and Q3), and the whiskers extending to the most extreme data point within 1.5× the interquartile range (IQR, from Q1 to Q3). TPM, transcripts per kilobase million.

**Figure S5 The antisense transcripts detected by RAG-seq2.0**

Tracks showing antisense transcripts *RNF213-AS1* and *ENSG00000290058* detected at the *RNF213* and *NUDT19* genomic loci by RAG-seq2.0 and NSR. Sense reads are shown in red, while antisense reads are depicted in blue. Arrows indicate the transcription direction (5'−3') for each RefSeq gene. NSR represents strand-specific bulk RNA-seq data generated using 1 μg of total RNA. The figure was visualized using the UCSC Genome Browser.

**Figure S6 Optimization of RAG-seq2.0 protocol**

**A.** Comparison of read coverage across gene body for RAG-seq2.0, RAG-seq3.0, SHERRY2, and Smart-seq2. The gray area indicates the standard deviation of normalized depth across replicates. **B.** Distribution of uniquely mapped reads across genome features in HEK293 cells with using varying amount of oligo dT primers. **C.** Number of genes (TPM > 1) detected with the RAG-seq3.0 protocol compared to RAG-seq2.0, Smart-seq2, and SHERRY2 in HEK293 cells. **D.** Pairwise correlation of gene expression within replicates for RAG-seq2.0, RAG-seq3.0, SHERRY2, and Smart-seq2. **E.** Correlation of gene expression between RAG-seq2.0 and RAG-seq3.0 with bulk RNA-seq (NEBNext) in HEK293 cells. For RAG-seq2.0 and RAG-seq3.0, 10 HEK293 cells were used (n = 3); SHERRY2 and Smart-seq2 published data from single HEK293 cells were downloaded from the NCBI SRA (PRJNA879104). Data in panels (B) and (C) are presented as mean ± SD. For box-and-whiskers plots in panels (D) and (E), the center line represents the median; the upper and lower lines of the box represent the first and third quartiles (Q1 and Q3), and the whiskers indicate the most extreme data points within 1.5× the interquartile range (IQR, from Q1 to Q3). *P* values in (D) and (E) were calculated by one-way ANOVA followed by Bonferroni’s multiple comparisons test (n.s., not significant; ***, *P* ＜ 0.001). NCBI, National Center for Biotechnology Information; SRA, Sequence Read Archive.

**Figure S7 The improvement of RAG-seq3.0 on Bulk RNA**

**A.** Distribution of uniquely mapped reads across genome features. **B.** Number of genes (TPM > 1) detected by RAG-seq2.0 and RAG-se3.0 using low-input HEK293 total RNA. **C.** Pairwise correlation of gene expression within replicates. **D.** Correlation of Gene expression between low-input HEK293 total RNA (10 ng and 1 ng) and bulk RNA extracted from HEK293T cells (NEBNext). **E.** Read coverage across the gene body for RAG-seq2.0 and RAG-se3.0. The gray area indicates the standard deviation of normalized depth across replicates. For RAG-seq2.0 and RAG-seq3.0, 10 ng and 1 ng HEK293 total RNA were used (n=3). Data in (A) and (B) are presented as mean ± SD. For box-and-whiskers plots in (C) and (D) show the median as the center line, first and third quartiles (Q1 and Q3) as the upper and lower bounds of the box, and the whiskers extending the most extreme data point within 1.5× the interquartile range (IQR, from Q1 to Q3). *P* values were calculated by two-tailed unpaired *t*-test (***, *P* ＜ 0.001).

**Figure S8 Transcriptome analysis in mouse early embryos**

**A.** Distribution of uniquely mapped reads across genome features. The percentage of reads aligned to coding, UTR, intronic, and intergenic regions. **B.** Number of genes (TPM >1) detected at each developmental stage by RAG-seq3.0 and Smart-seq3. **C.** Pairwise correlation of gene expression between replicates at the same developmental stages of early mouse embryos. **D.** Read coverage across the gene body for RAG-seq3.0 and Smart-seq3. The gray area indicates the standard deviation of normalized depth across replicates. **E.** Principal component analysis of mouse embryos using RAG-seq3.0 and Smart-seq3 based on gene expression. Data in panels (A) and (B) are presented as mean ± SD. For RAG-seq3.0, n = 4 per stage; For smart-seq3, n = 8 or 6 per stage for Smart-seq3. For box and whiskers plots in panel (C) show the median as the center line, first and third quartiles (Q1 and Q3) as the upper and lower bounds of the box, and whiskers extending to the most extreme data point within 1.5× the interquartile range (IQR, from Q1 to Q3). UMI and Internal represent the 5 UMI-containing reads and internal reads of Smart-seq3. PC1, principal component 1; PC2, principal component 2; UTR, untranslated region; UMI, unique molecular identifier.

**Figure S9 Antisense transcripts analysis of mouse early embryos**

**A.** Venn diagram showing the antisense transcripts detected by RAG-seq3.0 and Smart-seq3 in mouse early embryos. **B.** Tracks showing antisense transcripts detected at the *Dnajc2* and *Rnf10* genomic loci by RAG-seq3.0 and Smart-seq3. Sense reads are depicted in red, while antisense reads are shown in blue. Antisense transcripts in genomic regions are marked in shadow. Arrows indicate the transcription direction (5' −3') for each RefSeq gene. The 5’ UMI-containing reads of Smart-seq3 was used for analysis.

**Table S1 qPCR primers in this study**

**Table S2 The summary of primer and oligo sequences**

**Table S3 sgRNA sequence**
