# Supplementary figures and images for: RAG-seq: NSR-primed and Transposase Tagmentation-mediated Strand-specific Total RNA Sequencing in Single Cells
Source: Genomics Proteomics Bioinformatics. 2024 Oct 10;22(5):qzae072. doi: 10.1093/gpbjnl/qzae072 (PMC11658833; doi:10.1093/gpbjnl/qzae072)

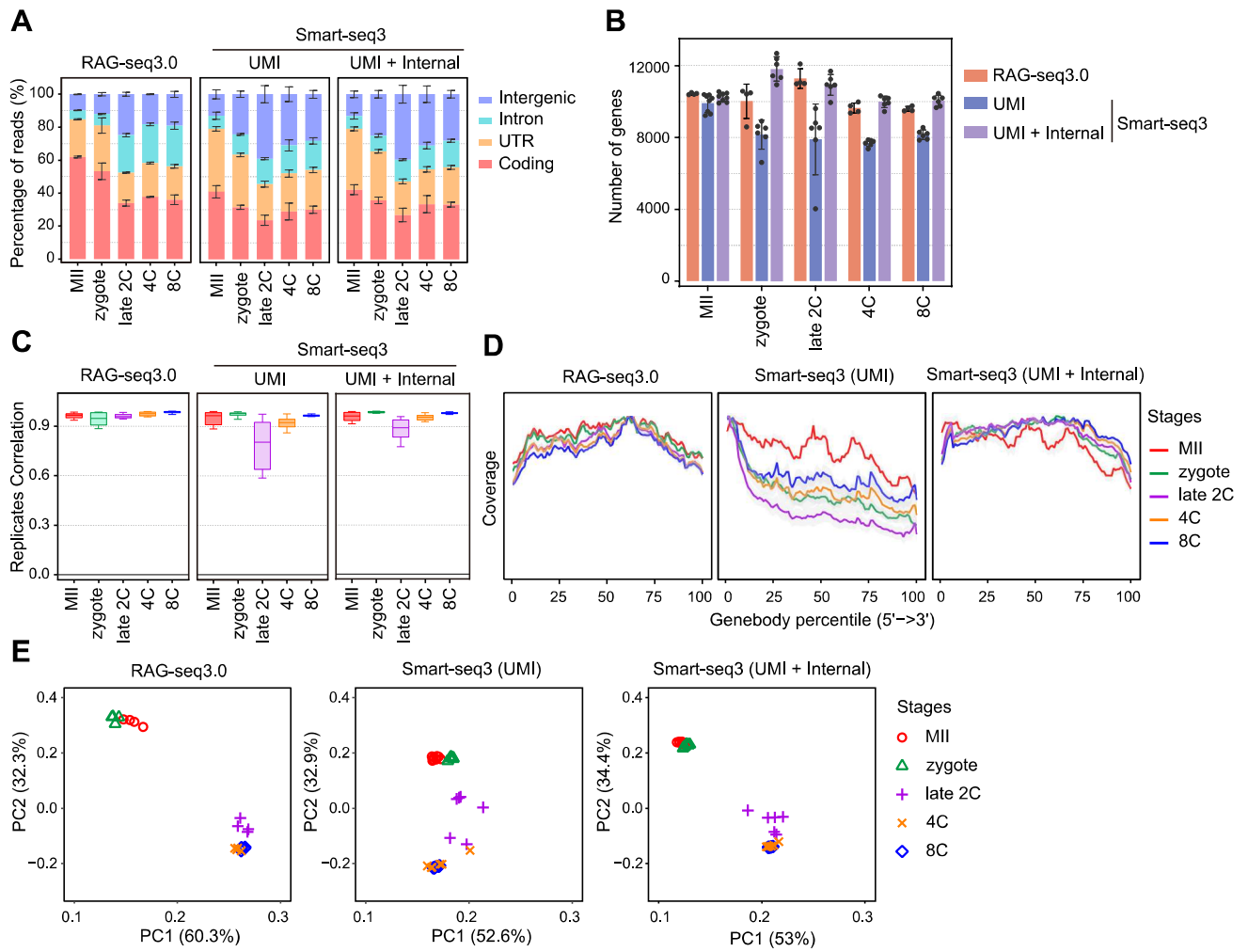

Supplement: qzae072_Supplementary_Data [file qzae072_supplementary_data.zip › Figure S8.pdf]

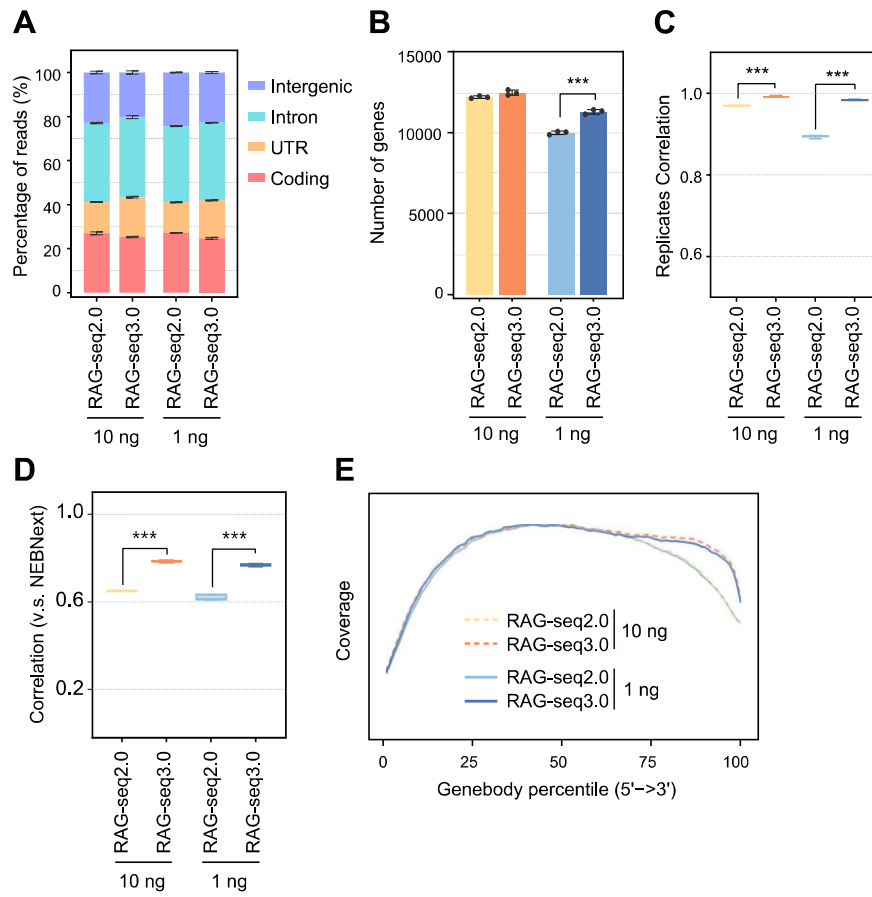

Supplement: qzae072_Supplementary_Data [file qzae072_supplementary_data.zip › Figure S7.pdf]

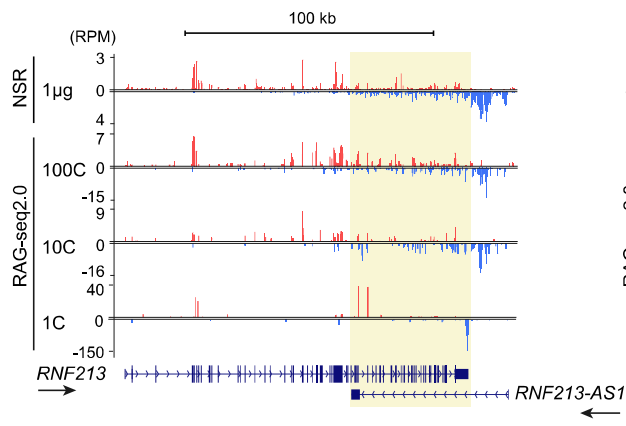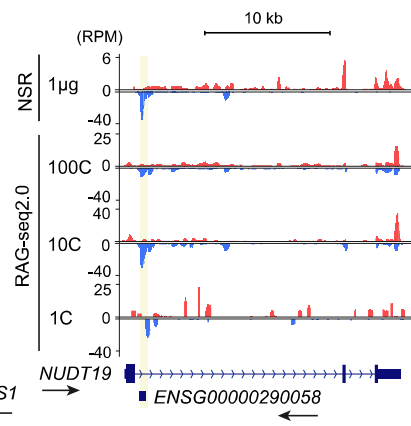

Supplement: qzae072_Supplementary_Data [file qzae072_supplementary_data.zip › Figure S5.pdf]

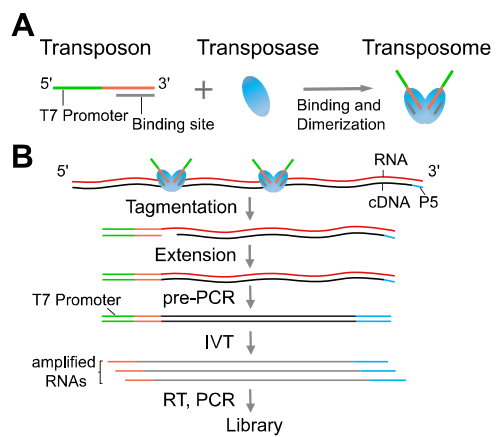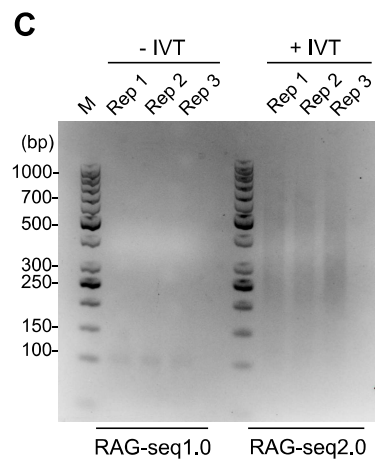

Supplement: qzae072_Supplementary_Data [file qzae072_supplementary_data.zip › Figure S1.pdf]

**A**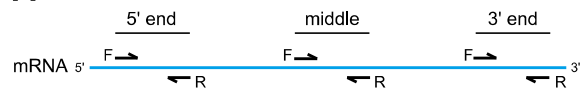

Reverse Transcriptase: SSII, GIII, SSIV, Maxima H

**B**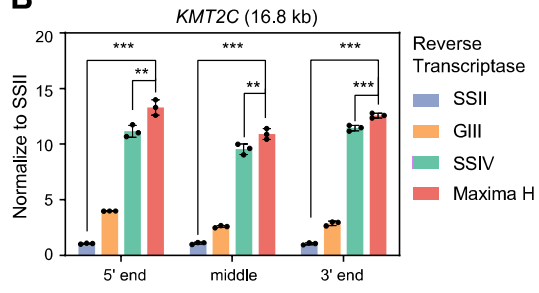

Supplement: qzae072_Supplementary_Data [file qzae072_supplementary_data.zip › Figure S3.pdf]

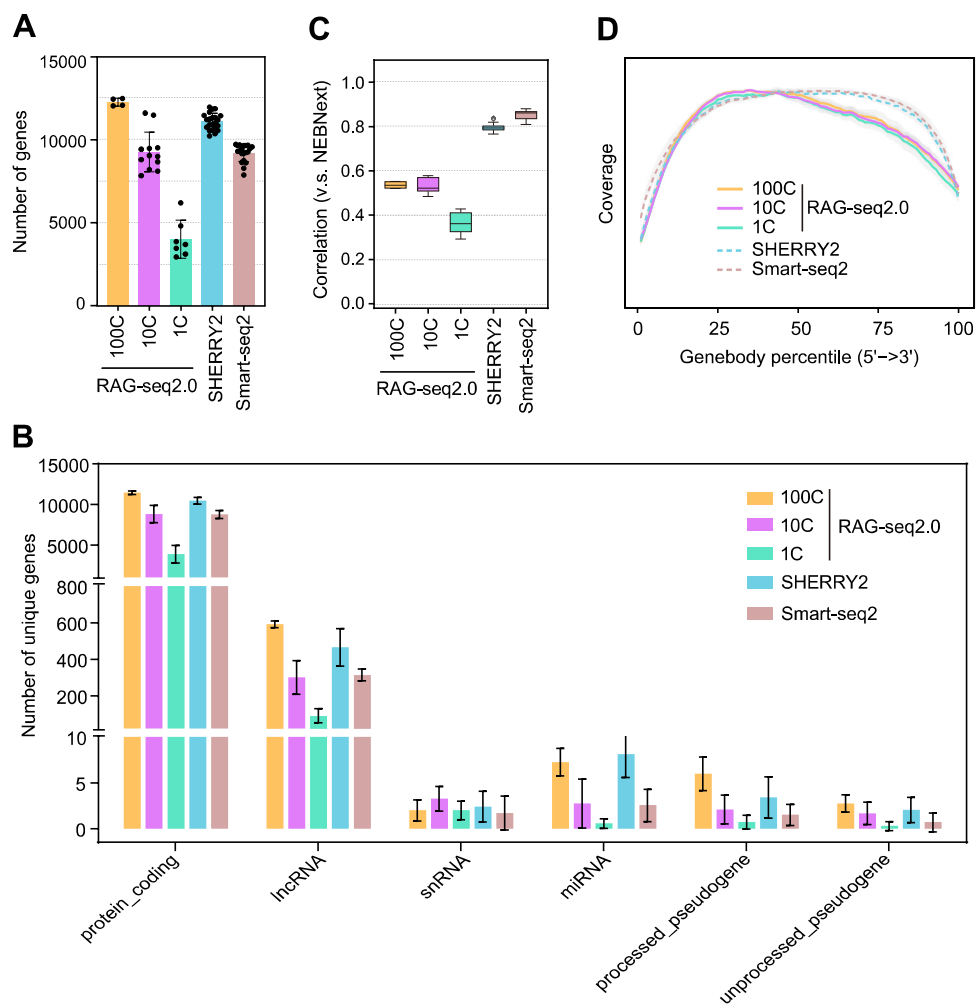

Supplement: qzae072_Supplementary_Data [file qzae072_supplementary_data.zip › Figure S4.pdf]

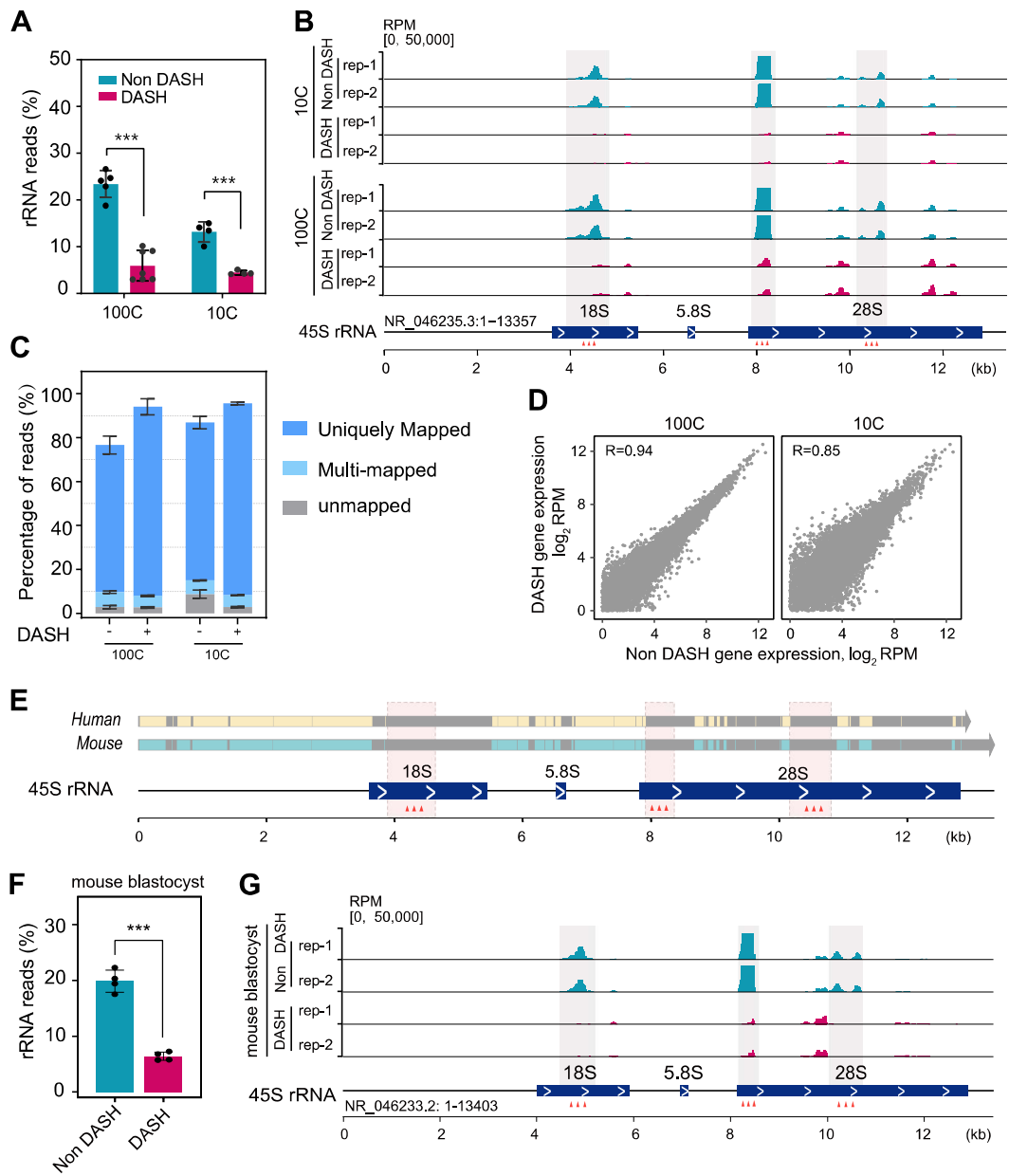

Supplement: qzae072_Supplementary_Data [file qzae072_supplementary_data.zip › Figure S2.pdf]

**A**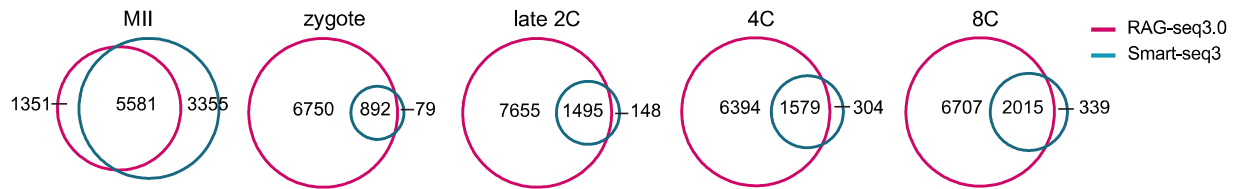**B**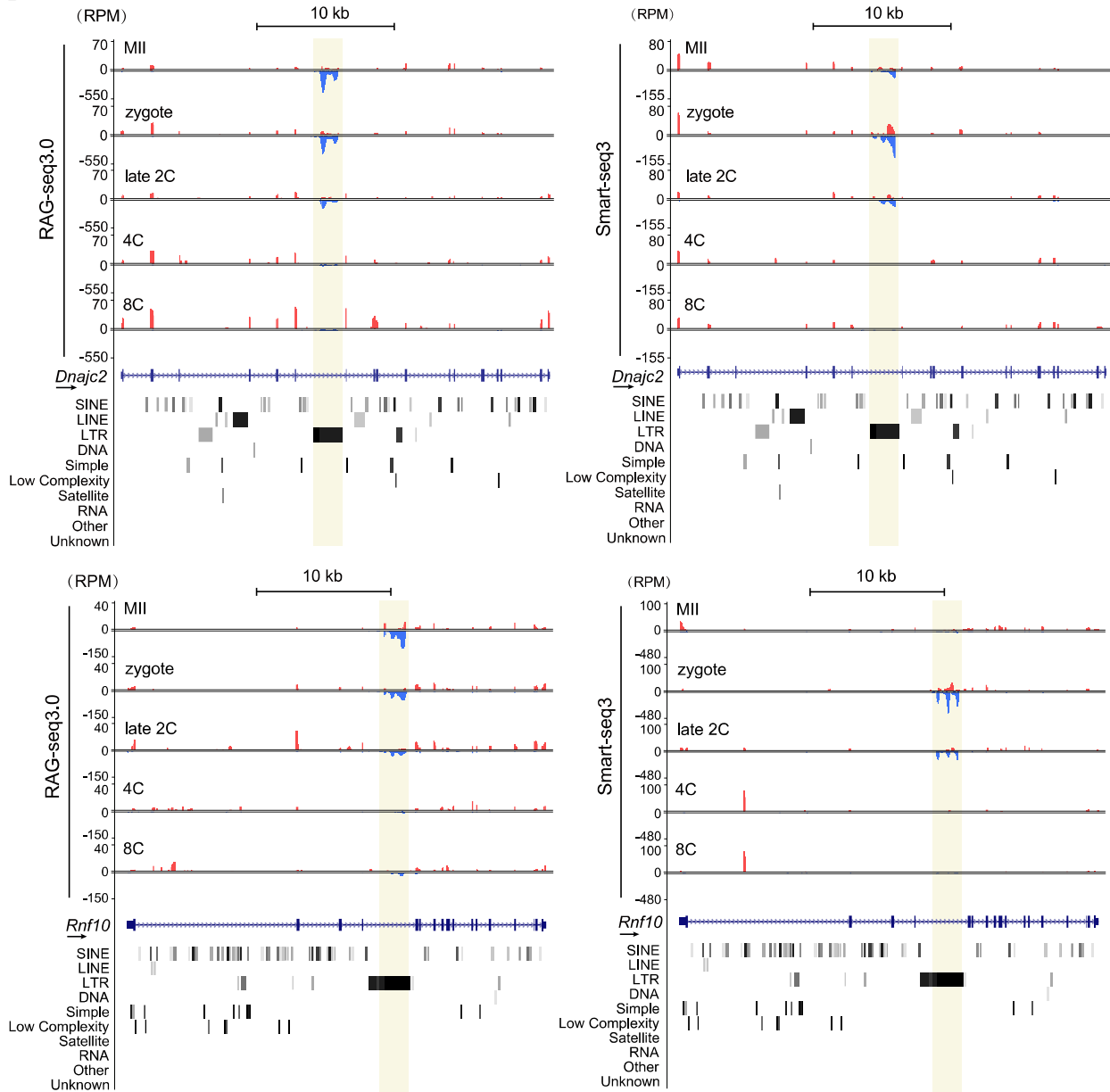

Supplement: qzae072_Supplementary_Data [file qzae072_supplementary_data.zip › Figure S9.pdf]

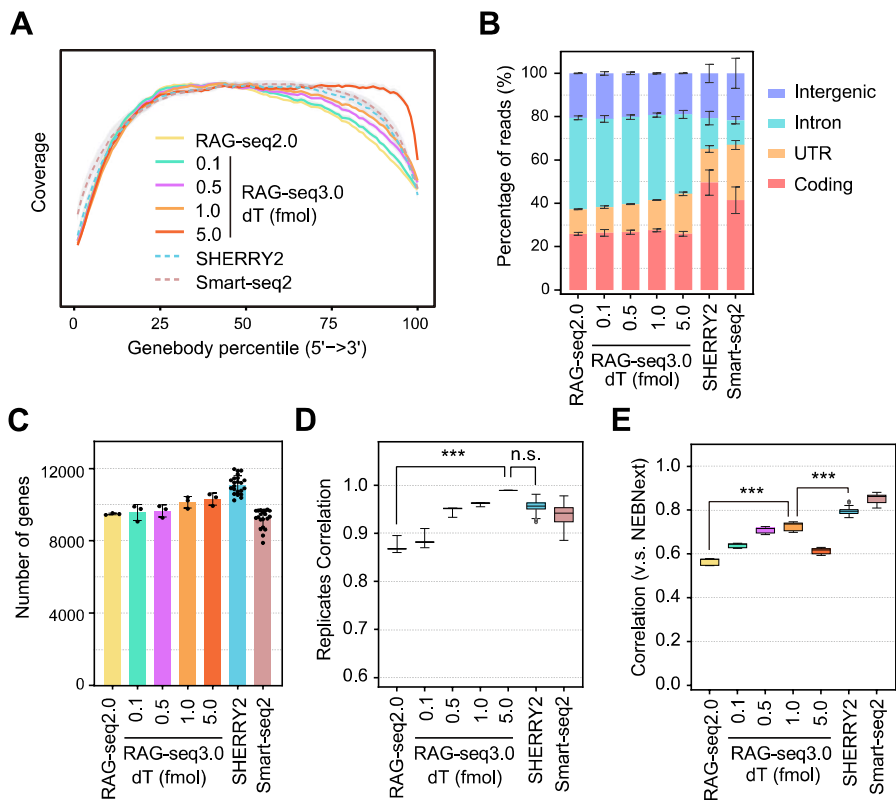

Supplement: qzae072_Supplementary_Data [file qzae072_supplementary_data.zip › Figure S6.pdf]
